# Supplementary material for: Whole Blood as a Sample Matrix in Homogeneous Time-Resolved Assay—Förster Resonance Energy Transfer-Based Antibody Detection
Source: Diagnostics (Basel). 2024 Mar 29;14(7):720. doi: 10.3390/diagnostics14070720 (PMC11011549; doi:10.3390/diagnostics14070720)
Supplement: Supplementary file 1 [file diagnostics-14-00720-s001.zip › diagnostics-2895704-supplementary.pdf]

Supplemental Table S1

Correlation (and R^2) of IgG and patient WB by months after infection

	10 min	20 min	30 min	40 min	50 min	60 min	Total
**All**	0.49 (0.24)	0.5 (0.25)	0.51 (0.26)	0.51 (0.26)	0.51 (0.26)	0.52 (0.28)	0.49 (0.24)
**<30 days**	0.43 (0.19)	0.38 (0.15)	0.34 (0.12)	0.43 (0.18)	0.34 (0.11)	0.39 (0.15)	0.32 (0.1)
**1-6 months**	0.57 (0.32)	0.54 (0.29)	0.54 (0.29)	0.52 (0.27)	0.57 (0.33)	0.56 (0.32)	0.53 (0.28)
**6-10 months**	0.59 (0.35)	0.61 (0.37)	0.59 (0.35)	0.57 (0.33)	0.57 (0.33)	0.59 (0.34)	0.56 (0.32)
**10-14 months**	0.38 (0.15)	0.49 (0.24)	0.46 (0.21)	0.47 (0.22)	0.48 (0.23)	0.49 (0.24)	0.45 (0.2)
**>14 months**	0.13 (0.02)	0.16 (0.03)	0.15 (0.02)	0.18 (0.03)	0.17 (0.03)	0.19 (0.04)	0.16 (0.02)

Correlation (and R^2) of neutralization titre and patient WB by months after infection

	10 min	20 min	30 min	40 min	50 min	60 min	Total
**All**	0.91 (0.82)	0.92 (0.85)	0.92 (0.86)	0.91 (0.83)	0.91 (0.84)	0.92 (0.85)	0.88 (0.78)
**<30 days**	0.33 (0.11)	0.42 (0.18)	0.44 (0.19)	0.55 (0.3)	0.42 (0.18)	0.56 (0.32)	0.39 (0.15)
**1-6 months**	0.94 (0.88)	0.94 (0.88)	0.93 (0.87)	0.94 (0.88)	0.93 (0.87)	0.93 (0.87)	0.9 (0.81)
**6-10 months**	0.93 (0.87)	0.94 (0.87)	0.95 (0.9)	0.93 (0.87)	0.93 (0.86)	0.94 (0.89)	0.9 (0.81)
**10-14 months**	0.93 (0.87)	0.93 (0.86)	0.95 (0.9)	0.96 (0.91)	0.94 (0.88)	0.95 (0.9)	0.9 (0.81)
**>14 months**	0.82 (0.68)	0.86 (0.74)	0.87 (0.75)	0.84 (0.7)	0.86 (0.73)	0.86 (0.74)	0.8 (0.64)

Correlation (and R^2) of IgG and patient WB by latest known vaccination status

	10 min	20 min	30 min	40 min	50 min	60 min	Total
**1-8 weeks**	-0.35 (0.12)	-0.38 (0.14)	-0.33 (0.11)	-0.31 (0.09)	-0.31 (0.1)	-0.28 (0.08)	-0.29 (0.09)
**9-16 weeks**	0.04 (0)	-0.01 (0)	0.05 (0)	0.11 (0.01)	0.05 (0)	0.13 (0.02)	0.06 (0)
**17< weeks**	0.35 (0.12)	0.39 (0.15)	0.37 (0.14)	0.39 (0.15)	0.39 (0.15)	0.39 (0.15)	0.37 (0.13)
**NA**	0.45 (0.2)	0.45 (0.2)	0.44 (0.19)	0.44 (0.19)	0.46 (0.21)	0.46 (0.21)	0.43 (0.19)
**Vaccinated later**	0.44 (0.19)	0.44 (0.2)	0.42 (0.17)	0.42 (0.18)	0.41 (0.17)	0.42 (0.17)	0.4 (0.16)

Correlation (and R^2) of neutralization titre and patient WB by latest known vaccination status

	10 min	20 min	30 min	40 min	50 min	60 min	Total
**1-8 weeks**	0.75 (0.56)	0.74 (0.55)	0.81 (0.65)	0.87 (0.76)	0.81 (0.66)	0.84 (0.7)	0.74 (0.55)
**9-16 weeks**	0.54 (0.29)	0.47 (0.23)	0.62 (0.38)	0.68 (0.46)	0.58 (0.34)	0.63 (0.4)	0.53 (0.28)
**17< weeks**	0.72 (0.51)	0.73 (0.54)	0.81 (0.66)	0.83 (0.68)	0.79 (0.63)	0.83 (0.69)	0.75 (0.57)
**NA**	0.91 (0.83)	0.9 (0.81)	0.93 (0.86)	0.94 (0.89)	0.92 (0.85)	0.94 (0.89)	0.89 (0.8)
**Vaccinated later**	0.89 (0.79)	0.93 (0.87)	0.91 (0.84)	0.94 (0.89)	0.91 (0.83)	0.94 (0.88)	0.88 (0.77)
